# Teachers’ Use of Within-Class Ability Groups in the Primary Classroom: A Mixed Methods Study of Social Comparison

**DOI:** 10.3389/fpsyg.2021.728104

**Published:** 2021-12-06

**Authors:** Jane Louise Webb-Williams

**Affiliations:** Education Futures, University of South Australia, Adelaide, SA, Australia

**Keywords:** ability groups, social comparison, self-evaluation, academic performance, teachers, engagement

## Abstract

It is common practice within primary classrooms for teachers to spilt children into different ability groups so that children of similar level are taught together. Whilst this practice is used across the globe, research is mixed on the benefits of such grouping strategy. This paper presents data collected from mixed methods research which investigated teachers use of grouping strategies and social comparison, the act of comparing oneself with others. It focuses on when, why and with whom children from different ability groups compare themselves and the impact this has on their self-perceptions. Drawing upon data from children aged between 10 and 11 years from 12 primary schools, social comparison was found to play a significant role in daily classroom life for some children. The study identified different strands of the social comparison process including acknowledgment, topic, target, and direction, and it revealed positive and negative effects of social comparison. A difference by ability group was identified. Children within the low ability group were particularly vulnerable to the negative effects of social comparison and found to engage in more frequent and intentional social comparisons which were heavily relied upon for self-evaluation and performance evaluation. The paper discusses the educational implications of social comparison regarding pupil ability grouping strategies, motivation, engagement, and academic performance. Implications for teacher education and professional development is discussed.

## Introduction

Many educational systems utilize grouping by ability in which children of similar attainment level are taught together and this practice is increasing internationally (Taylor et al., 2020). In England, grouping by ability is a highly prevalent practice in secondary schools (Taylor et al., 2020) and increasingly common in primary schools where it is encouraged by school leaders and seen as expected practice by teachers (Bradbury and Holmes, 2017). Some authors have suggested that this trend may reflect pressures on teachers to raise standards and meet targets (McGillicuddy and Devine, 2018; Bradbury et al., 2021), however, the research for such positive outcomes of grouping is limited. Previous research on the motivation for grouping found that teachers like ability groups (Hallam and Ireson, 2007), pointing to the ease of teaching children of similar level of attainment in terms of differentiation, behavior management, and classroom management (Muijs and Reynolds, 2005). While these reasons for grouping still prevail recent research suggests that teachers are conflicted about their use of grouping practices and concerned about its impact (Bradbury and Holmes, 2017; McGillicuddy and Devine, 2018). The research evidence appears to be at best mixed on the benefits of ability grouping strategies (Hallam et al., 2004) and at worst ‘detrimental’ (Liem et al., 2013; Taylor et al., 2020) or even ‘harmful’ (Boliver and Capsada-Munsech, 2021), with researchers highlighting that there is little or no evidence for ability groups (Hattie, 2002, 2009; Marsh et al., 2014). A key issue is that grouping by ability is not simply based on attainment but rather formed through assumptions, bias and judgment by teachers which can lead to exclusion and in equality. Thus, grouping pupils by ability remains controversial (Hallam and Parsons, 2013; Francis et al., 2017).

Ability grouping strategies tend to involve either allocating children into *different* classes or allocating children to different groups within the *same* class. Grouping children into *different* classes is referred to as ‘streaming’ or ‘setting.’ Streaming’ is where children are grouped across classes in a year level according to current attainment and this grouping remains constant across subjects. ‘Streaming’ therefore means that children are taught in the same group (‘stream’) for all/most of their subjects. ‘Setting’ involves allocating children to different ability ‘sets’ for different subjects, e.g., Mathematics and English, but this often doesn’t involve all subjects across the curriculum. In contrast to setting and streaming, within-class ability grouping involves forming groups of children within their usual class for specific subjects or activities. Children of similar attainment levels are grouped together and tend to be allocated to specific tables so they sit/work together in their group. Within-class ability grouping means that all children within the class are taught by the same class teacher and tend to follow the same curriculum. Children in the different groups are given different levels of challenge, expectations, and support. The teacher may use different ability groupings for different topics, tasks, activities as well choose to utilize mixed-ability groupings where children are selected from different ability levels to form groups.

Pitfalls associated with the practice of streaming and setting have been identified including the negative impact on children’s performance and self-concept (Marsh, 1984b; Oakes, 1985; Slavin, 1987; Liem et al., 2013; Taylor et al., 2020) and although within-class ability groupings are common place in English primary schools (schools catering for children aged 5–11) some research has suggested there is a potential for negative effects on pupils assigned to the low-ability groups (MacIntyre and Ireson, 2002; Muijs and Reynolds, 2005; Boliver and Capsada-Munsech, 2021). This calls for teachers to consider the impact of the grouping strategies they are employing and the way they are assigning children to ability groups, yet research points to the arbitrary, unplanned, and less than satisfactory way groups are formed within primary schools.

Measures of prior/current performance are used by teachers to form groups and thus ‘achievement’ rather than ‘ability’ is sometimes used to refer to these groupings. That said, the assumption that groups are based solely on measures of attainment is far from accurate. Muijs and Dunne (2010) examined how teachers decide upon ability groups and found that, although they were based to a large part on actual prior attainment, teachers were also influenced by factors such as special educational needs and social class. Thus, teachers add their own criteria to decide which ability group children should be allocated. This illustrates the subjective nature of grouping mechanisms and as such there is a danger of exclusion and inequality whereby teachers are forming groups on their own opinions (Campbell, 2014). Furthermore, given groupings are to a large extent imposed by the individual classroom teachers, children (i.e., the group members) tend to have no input into the formation, implementation or functioning of the groupings. Indeed, Kutnick (1990, p. 119) suggests that peer relations are defined by the “society and structured interactions” in which children are allowed to participate and the lack of choice and lack of empowerment for children in the decision-making process has implications for group interactions.

Social interactions in the classroom have long been known to be important for student motivation, learning, and academic performance (Juvonen and Wentzel, 1996; Buhs and Ladd, 2001). Children typically spend significant time interacting with their peers (Dijkstra et al., 2011), particularly near-seated peers (Van den Berg et al., 2012), and research shows the importance of classmates and friendships (Rambaran et al., 2017; Maunder and Monks, 2019) and liked-minded peers for belonging (Riley and White, 2016). Indeed, previous research has suggested that children spend more time interacting with their peers than in one to one or small group interactions with their teachers (Galton, 1989). Moreover, with the focus on collaborative group work and peer-led tasks in contemporary classrooms (Siraj-Blatchford, 2007; van Drie and Dekker, 2013), pupil–pupil interactions play an increasing role in teaching and learning.

Research on pupil–pupil interaction focusing on social comparisons (the act of comparing oneself with others) show that children compare their own achievements, abilities, or characteristics with that of their peers which leads them to feel more positive or negative about their own competencies. Such judgments of self in relation to others can have a significant impact on children’s performance (Blanton et al., 1999; Huguet et al., 2001), self-evaluations (Frey and Ruble, 1985; Crabtree and Rutland, 2001), self-efficacy beliefs (Bandura, 1997; Webb-Williams, 2018), and a large body of work on the Big Fish Little Pond Effect (BFLPE) conducted by Marsh and colleagues (e.g., Marsh, 1984b; Marsh et al., 1995; Marsh and Hau, 2003) has shown social comparisons to be central to the formation of academic self-concept.

The classroom environment is the perfect context for social comparisons to occur as it provides an abundance of comparative information, as pupils work together and share ideas, allowing observation of information about peers including assignments, classwork, grades, discussions as well as teacher feedback. We know that this social comparative information is used by children to judge themselves, but questions remain about the underlying process. How is the social comparative information in the classroom experienced by children? Do children of different levels of attainment interpret the information differently? What impact does peer comparisons have on children’s thoughts, feelings and behaviors and how are these acted out in the classroom environment? The answers to these questions are pivotal to our understanding of social comparison processes in the classroom. If we can provide teachers with information to help them support social comparison, we can potentially create positive outcomes for children. This is particularly salient because when grouping by ability teachers instruct children to work together or sit together in groups giving little choice to the child over who they interact. This means that teachers have the power to alter pupil–pupil interaction and thus directly impact children’s social comparison processes. This raises questions regarding whether teachers plan for pupil–pupil interaction or whether they only plan for their own interactions with pupils. Previous research examining classroom groupings suggest that groups tend to be formed by teachers with little regard to the “social pedagogic potential” of the group (Blatchford et al., 2003, p. 156) and thus issues such as size, composition and pupil–pupil interaction, and friendships are often ignored (Gremmen et al., 2018).

To date social comparison, despite its long history of research, doesn’t appear to be a strong feature of educational practice. Some have suggested that inconsistent findings in research has made it difficult to provide useful suggestions for educational practice yet it is argued here that the reliance on large scale quantitative laboratory studies means that the findings are difficult to contextualize in real world classroom settings. Some authors have raised caution over generalization of forced hypothetical measures of social comparison given differences in findings between field studies and laboratory studies (Dijkstra et al., 2008; Gerber et al., 2018; Boissicat et al., 2020). Qualitative research could provide the missing contextual understanding and richness of data.

This paper addresses this gap by qualitative and quantitative data to explore the nature of social comparison in within-class ability groups in English primary schools. The aim is to understand the ways in which social comparison is experienced by children within different ability groups. If we can gain more information on how this powerful construct works in the classroom environment, we will be better placed to arm teachers with information to support ability grouping decision making.

### Social Comparison Theory

Social comparison theory, originally proposed by Festinger (1954), is concerned with the processes involved in comparing ourselves with others. Social comparison, therefore, can be seen as an examination of the accuracy of one’s self-beliefs and attitudes. In this way social comparison theory encapsulates an important aspect of human social life; that other people provide the standards against which one can judge oneself. Wood (1996) furthers this definition of social comparison as the process of thinking about information about one or more other people in relation to the self (p. 521). In this definition Wood (1996) expands the meaning of social comparison to incorporate comparisons with stereotypes and hypothetical characters.

People seek social comparison when it is not possible to base self-evaluations on objective non-social criteria, preferring to compare themselves with people that they identify as being similar to themselves. In this way social comparison leads toward uniformity, that is, a difference of opinions or abilities tends to be accompanied by a change in oneself to be like others or change in others to be like oneself. Extending this theory, Brickman and Bulman (1977) showed that people not only seek social comparisons but also *avoid* them. In seeking social comparisons people gain valuable information and learn through observation of others. This “adaptive” consequence of social comparison contrasts with the “hedonic” consequences such as preservation of well-being which occur when people avoid social comparisons. Relating social comparison theory to the classroom context, it is easy to see why children may try to *seek* or *avoid* comparisons with particular peers and why knowledge of this, together with the knowledge that social comparisons can occur at the intragroup, intergroup and personal level (Guimond, 2006), would be important to teachers forming groups.

#### Social Comparison in the Classroom

In their review of social comparison in the classroom, Dijkstra et al. (2008) note the long history of social comparison research, yet the multiple transformations of the theory from Festinger’s (1954) original work. They highlight the move away from self-evaluation as the only motive for social comparison but that individuals hold many reasons for social comparison such as self-enhancement and self-improvement (Wood, 1989; Collins, 2000; Dijkstra et al., 2008). Moreover, over the past 40 years the research literature has provided strong evidence that social comparisons operate at multiple different levels within educational settings.

The Big Fish Little Pond Effect (BFLPE, Marsh, 1984a, Marsh, 1987; Trautwein et al., 2009; Liem et al., 2013) has shown that students of comparable ability do better in lower-achieving classes or schools than in higher-achieving classes or schools. The understanding here is that children evaluate themselves more favorably against low-ability others than comparing to higher performing others which can lead to negative feelings about their own ability. Thus, it is better for a student’s self-evaluation and academic self-concept to be a big fish in a large pond than a small fish in a big pond. Considerable evidence of this phenomenon has shown it to be robust internationally across gender and culture, arguing it be a result of social comparison (Marsh, 1987).

While BFLPE operates due to social comparisons, inconsistencies with Social Comparison Theory led researchers to call for direct evidence of the social comparison processes within BFLPE (Dai and Rinn, 2008). Following the work by Huguet et al. (2009) BFLPE was shown to be routed in how students compare with their school or class taken as a whole. School or class level comparisons in the BFLPE operate by using a generalized frame of reference where individuals make comparisons to the average/general ability of their class or school. School/class comparisons can operate independently to local social comparisons which operate at the individual level using one or a few people in the immediate environment as the frame of reference. The Local Dominance Effect (Buckingham and Alicke, 2002; Zell and Alicke, 2010) has shown that local comparisons with a small number of individuals has a stronger influence on self-evaluations than general comparisons with larger aggregates.

In addition to group and individual social comparisons, certain key elements of social comparison research appear to be significant when considering the decisions teachers make about ability-grouping namely are discussed below: (1) the negative and positive impact of social comparison direction and target and (2) social comparison and self-evaluation, and (3) the relationship between social comparison and performance.

#### Social Comparison and Self-Evaluation

Social comparisons have long been shown to be pivotal to self-evaluations. Crabtree and Rutland (2001) found that self-perceptions of competence changed significantly when the social comparative context was artificially altered. In the primary school classroom, the teacher may alter the social comparative context regularly by instructing children to work together in different groups for different subjects or activities. In doing so teachers alter children’s self-evaluations, knowingly or unknowingly. The issue is that self-evaluations based on social comparison and normative information can have a negative impact self-evaluations and academic behaviors, e.g., effort and engagement and some research has suggested that this negative impact is particularly acute for low achievers (Levine, 1983). This may indicate that disadvantaged groups are more vulnerable to the threat of comparisons and that they use strategies to protest themselves from this threat. Indeed, self-evaluation maintenance theory (Tesser, 1988) assumes that all individuals try to maintain a positive self-evaluation and utilize alternative ways in which social comparative information is received and processed. According to self-evaluation maintenance theory, by altering ones performance, the relevance of the domain or the closeness of the comparison target one can protect one’s self-evaluation. The more important or relevant a domain to one’s identity the more likely one will suffer a lower self-evaluation. For example, if one attaches a high value to a particular domain and as such it is viewed as part of the individuals identity then that individual will hold a lower self-evaluation when a close friend achieves a higher accolade.

#### Direction and Target of Social Comparison

Previous research has tended to focus on who people seek to compare themselves with, the ‘social comparison target’ (SCT). For example, research conducted over two decades ago found that children at secondary school compare themselves upwards with a comparison target that slightly outperforms them (Blanton et al., 1999; Huguet et al., 2001). Research like this has provided many valuable insights regarding the affective consequences of social comparison and the direction of social comparison. The direction of social comparison (upwards, downwards, or horizontal) is a key concept within social comparison theory, e.g., Suls and Wheeler (2000). People who seek improvement make “upward” comparisons with people superior to themselves and people who seek preservation make “downward” comparisons with inferior others. However, researchers identified some time ago that social comparisons can result in positive and negative effects depending upon whether they are perceived as *contrasts* or *assimilations* (Mussweiler et al., 2004). *Contrasts* occur when individuals emphasize the differences between themselves and the comparison target. Making contrasts in downward comparisons is likely to produce feelings of superiority and confidence whereas making contrasts in upward comparisons tends to lead to feelings of jealousy and inadequacy. *Assimilations* can also have positive and negative consequences. When individuals make assimilations they emphasize their similarities to the comparison target. Thus, upward assimilations are associated with learning and growth as individuals aim to be more like the comparison target. However, making assimilations with downward comparisons has the opposite effect since individuals focus on the similarities between themselves and the lower comparison target leading to increased anxiety and self-doubt. The direction of comparison could therefore give us important information about how children feel when assigned to different ability groups. Indeed, upward comparisons between groups (intergroup) can reduce group identification and lower self-esteem (Smith et al., 1994). In contrast, downward intergroup comparison can enhance self-esteem and increase group identification (Martinot and Redersdorff, 2003, 2006). It should be noted that according to some researchers (e.g., Hogg, 2000) these orientations can co-occur depending upon personal motives such as self-enhancement or self-evaluation.

#### Social Comparison and Performance

Social comparison theory also has important implications for task performance and some research has suggested that upward comparisons can enhance performance. For example, Blanton et al. (1999) found that upward comparison predicted academic performance in secondary school, Levine (1983) reported that social comparison affects aspects of performance such as attention and persistence, Monteil (1988) found that the performance of adolescents differed according to the amount of social comparative information given, and Vrugt (1994) found that high comparative evaluations were associated with less negative feelings about school which were associated with better academic performance. That said, the impact of social comparison on measures of academic performance has not been systematically studied (Huguet et al., 2001). However, it has been established that children pay more attention to how their performance compares to that of their peers than to how their performance compares to their own past performance (Ruble and Flett, 1988; Gremmen et al., 2018) which suggests that perceptions of performance rather than actual performance is important. Thus, with its implications for social interaction, groupings, self-evaluations and academic performance social comparison research can provide important educational implications.

#### Measurement of Social Comparison

One issue within the field concerns measurement of the construct. Researchers such as Ruble (1983) and Harter (1996) have suggested that people are reluctant to admit to engaging in social comparison which provides a difficulty in obtaining valid responses from participants. As Buunk and Gibbons (2000) note, it is most likely that there are a variety of factors resulting in an individual’s reluctance to admit that they compare themselves with others. It may be a cognitive lack of awareness, an issue of social desirability and/or it may be partly dispositional.

Further limitations of social comparison measures are that they have tended to focus on forced hypothetical social comparisons These measures are forced because typically the researcher will present individuals with a task or a scenario, and then ask them to judge themselves relative to the performance of others on the task/scenario. Thus, individuals are forced to compare as they have no option but to answer, yet whether the individual would actually compare like this spontaneously is unknown. Thus, some researchers have cautioned about generalizing these findings and raise the issue that results from such designs may differ from studies examining everyday/spontaneous comparisons (Dijkstra et al., 2008; Gerber et al., 2018; Boissicat et al., 2020). Indeed, in a recent meta-analysis of 60 years of social comparison work Gerber et al. (2018) found differences in the findings from field studies to laboratory studies. Whilst reporting bias and potential halo effects of direct comparison measures (e.g., comparative ratings) may be small, there is an acknowledgment that these methods need more work to “evaluate their psychometric properties and construct validity in relation to social comparison processes” Marsh et al. (2014, p. 62). Moreover, forced comparisons conducted in a laboratory limit real world application (Wood, 2000), and researchers have therefore suggested employing a range of methods (Wood, 1996, 2000) and to introduce methods which reduce the social desirability effects (Light and Littleton, 1999). Moreover, measuring social comparisons that occur in daily life have been advocated to increase ecological validity and gain greater understanding of comparisons (Argio et al., 2019). While research of ‘everyday’ comparisons self-reports and daily diaries naturalistically have been conducted (Wheeler and Miyake, 1992; Möller and Husemann, 2006; Summerville and Roese, 2008; Argio et al., 2019) there remains a gap in the field as qualitative/mixed methods designs are still relatively rare in social comparison research within education.

## Aims of the Study

Given the research discussed above, the current study aimed to:

(1) To gain an understanding of the nature of social comparison within primary school.
(2) To understand the ways in which social comparison is experienced by children within different within-class ability groups.
(3) To explore the effects of social comparison on children’s self-evaluations.

To address these issues the present study employed a range of strategies to measure social comparison, focusing on social comparisons that occur in an everyday context in the natural classroom environment. By utilizing a mix of qualitative and quantitative methods the aim was to uncover the complexity of social comparisons in the classroom and provide rich descriptions of school children’s experiences of social comparison, thereby furthering our understanding of peer–peer interactions in ability groups.

## Methodology

Qualitative and quantitative data were collected together in a parallel mixed model design (Teddlie and Tashakkori, 2009; Creswell and Plano Clark, 2011). Following the guidelines of mixed method design (Creswell and Plano Clark, 2017) the aim of this approach was to gather quantitative measures of social comparison together with qualitative data of children’s experiences, explanations and understandings of social comparisons. The intent of the design was to gain a better understanding of social comparison in the classroom. Whilst the quantitative and qualitative measures weren’t administered at exactly the same time, the minimal time lapse between the methods was such that the design was considered concurrent (Creswell and Plano Clark, 2017). The rationale for the use of mixed methods was threefold: triangulation (convergence of results from different methods), complementarity (elaboration/illustration of results from one method to the other method) as well as expansion of the breadth of the research by using different methods. As Creswell and Plano Clark (2017) suggests, the design of the study needs to match the research questions and as such many mixed methods studies are not designed with equal importance in the quantitative and qualitative aspects. In practice, one method often drives the study and the other method is used to confirm, explain, describe or expand the data collected using the other method. In the current study, the qualitative aspect of the study was given a dominant status (Johnson and Onwuegbuzie, 2004). The reason for such status lies in the aims of the study which focus on understanding and exploration of children’s experiences of social comparison, and the extended (repeated) semi-structured interview data collection phase relative to the shorter quantitative phase. On considering the design matrix of mixed methods research the study can be categorized as ‘QUAL + quan’ (Johnson and Onwuegbuzie, 2004; Morse, 2009). The use of capitals denotes the priority of the qualitative aspect, the ‘+’ denotes the concurrent mixed methods design, and the lower case denotes the supplementary nature of the quantitative data. That said the qualitative and quantitative data were concurrently collected and analyzed separately before being integrated in the interpretation phase. Thus, considering priority, implementation, and integration in the mixed methods design and given that the research questions aimed to explore children’s experiences of social comparisons, the qualitative data in the present study was prioritized. Consistent with such mixed methods design (Zhang and Creswell, 2013; Creswell and Plano Clark, 2017) the integration of the qualitative and quantitative data (the mixing) occurred during the results and interpretation stage and are presented in the Results narrative.

### Participants and Procedures

Two hundred and forty-six children aged between 10 and 11 years (mean age 10.2 years; 117 girls, 129 boys) participated in the study. The large majority of children (94%) were identified as White British. Children attended twelve English primary schools educating children aged between 4 and 11 years. Schools varied in size and included small and large schools (roll size ranged from 138 to 667). No relationship existed between the researcher and the participating schools. Schools were chosen according to geographical location (all schools were located in East Anglia in the United Kingdom) and all schools were identified as employing within-class ability grouping. In each school one Year 6 class (children aged 10–11 years) was nominated by the Principal to participate in the study. All children in that class whose parent/guardian had provided consent completed a social comparison questionnaire (described below). Stratified random sampling was used to select children for interview according to gender (boys, girls) and teacher assigned within-class ability grouping (high, medium, low). Each class teacher provided details of the within-class ability-groupings. While some teachers used more than three groups, all teachers allocated children to groups to the most frequent group the child was assigned which they classified as low-ability, medium-ability, and high-ability. One child from each of the three ability-groupings (high, medium, low) was selected from each school. Prior to commencement of data collection, each school received a list with the selected children. At this point teachers were asked to confirm availability and to check ability group assignment. In instances where a child was unavailable (e.g., illness or timetabled events) the pupil was excluded, and another pupil selected. In instances where teachers indicated that the child was not a good fit in the assigned group (low, medium, high) for example when a child was assigned as ‘medium’ ability but was not medium across all core subjects (maths, science, and English) another child was selected. Thirty-six pupils (18 boys, 18 girls) were involved in the individual interviews, three children from each of the 12 schools. The researcher visited each school three times over a 12-week period, administering all questionnaires in the first visit, and conducting all interviews in visit two and three. The study was conducted in compliance with the British Educational Research Associations Ethical Guidelines and appropriate ethical approvals and consents where obtained.

#### Instruments: Social Comparison Measures

Previous research suggests that a number of ways of accessing social comparison should be employed (Wood, 1996; Gerber et al., 2018). The current study included three categories of measurement in the quantitative questionnaires and semi-structured interviews:

(1) the *selection approach* examined the information children sought when making social comparisons,
(2) the *reaction approach* examined the impact of social comparison,
(3) the *narration approach* examined children’s everyday social comparisons.

#### Quantitative Instruments: Social Comparison Questionnaires

Paper copies of the quantitative questionnaire were distributed by the researcher during the first visit to each school. To reduce potential misunderstanding of the topic, the researcher informed the children prior to the administration of the questionnaire that social comparison did not mean copying (see below). Children were asked not to discuss the questionnaire with others but to complete the questions on their own using pen or pencil. Children identified as needing support were helped with reading, understanding and/or writing by the researcher or teacher. Responses required an open written response, e.g., “Describe how you feel when your schoolwork is not as good as other peoples?” or required a response to a question using a Likert scale with content descriptors for each point for example ‘Do you compare your schoolwork with other people? (1 = Yes, 2 = No, 3 = Not sure). As mentioned above the social comparison questionnaire included questions across selection, reaction and narration categories.

##### Selection Approach: Quantitative

The techniques used with this study included a modified version of the rank order paradigm developed in seminal work by Wheeler (see Wheeler, 1991) and comparison target (Blanton et al., 1999). In t*he rank order paradigm* participants were presented with information about the rank order of a hypothetical test. The children did not actually complete the test, as in the original paradigm. Instead they were asked to imagine a scenario in which they had been given the results of a test and asked to imagine they held the middle rank. Children were then asked to select one rank whose score they would like to know (1 = the top, 2 = just below top, 3 = above middle, 4 = just below middle, 5 = the bottom). In this way direction of comparison (up or down) was assessed. Next *SCT* was assessed using a free choice approach (Huguet et al., 2001) where children were asked to respond to questions about the pupil with whom they typically compare. Children were asked to circle the most appropriate. (1 = They are good at the subject, 2 = They sit next to me, 3 = They are the best in the class at the subject, 4 = They are bad at the subject, 5 = They sit next to me, 6 = They are the worst in the class at the subject, 7 = They help me, 8 = Other). Participants then described how good their SCT was compared with other classmates (1 = much worse, 2 = a little better, 3 = the same, 4 = a little better, 5 = much better).

##### Reaction Approach: Quantitative

To examine the impact of social comparisons, questions focused on affective responses to positive and negative assessments in relation to others. Participants described how they felt when their schoolwork was not as good as, or better than others. Responses were coded as 1 = positive, 2 = negative, 3 = neutral.

##### Narration Approach: Quantitative

The narration approach to measurement of social comparison refers to methods in which individuals report the comparisons they make during their everyday lives. Participants were asked whether they compared their schoolwork with others (1 = Yes, 2 = No, 3 = Not sure), how often they compared (1 = Never, 2 = Not often, 3 = every week, 4 = every day, 5 = All the time) and asked what subject they tend to compare (1 = Maths, 2 = English, 3 = Science, 4 = Sport, 5 = Other).

#### Qualitative Instruments: Semi Structured Interviews

The general aim was to understand children’s experience of social comparison. Interviews were semi-structured and were guided by the children’s responses. This ensured that pupils could freely explore, discuss and describe issues, observations and self-reflections as they desired. Every child was asked a similar set of semi-structured interview questions. As recommended by Tashakkori and Teddlie (1998), the funnel interview was used since it is directly applicable to mixed methods research. In this type of interview, the researcher mixes open and closed-ended questions, starting with broad questions and ending with focused issues. Interviews, which were conducted on school premises, tended to last no longer than 20 min and all responses were audio taped. All interviews included some ‘get to know you’ questions in order to build rapport between the researcher and participants. In order to remove potential issues due to social desirability and ensure children understood the terms used, any questions that directly asked about social comparison were accompanied with clarification of social comparison as opposed to copying. It was important that the children did not confuse comparison with copying. The latter is frowned upon in a school environment and thus any questions regarding this would potentially produce socially desirable answers rather than what children truly think, feel and behave. Of the 36 pupils interviewed, 12 pupils were in the “high” group, 12 pupils in the “low” group and 12 pupils in the “medium” group. Thus, an even number of pupils were interviewed at each ability group. Children were interviewed twice by the same researcher, with a 3-week break between interviews. The interviews were guided by children’s responses and covered all three approaches to social comparison measurement (discussed above). The list below gives an indication of the questions asked:

• Do you compare your schoolwork with other children?
• Why do you compare and how often?
• What subject do you tend to compare more in?
• Why do you think you compare more in this subject?
• Who do you compare you work with and why this person?
• How does it make you feel when you compare with this person?
• How good are you at school subjects compared with this person?
• Describe what makes you think this.
• How good at work is this person compared with others in the group/class?
• What makes you think this?
• How good are you at school subjects compared to the others?
• How can you tell?
• How does it make you feel when you compare against someone who is better than you?
• How does it make you feel when you compare against someone who is worse than you?

### Analytic Approach

The general aim was to understand children’s experience of social comparison. Audio tapes of the interviews were transcribed. Transcriptions and field notes were then analyzed using open coding to identity themes and look for patterns (Miles and Huberman, 1994). Inductive and deductive approaches to analysis were utilized and coding involved two levels, a data driven level and a theoretically driven level. For the first level coding social comparison theory provided theoretical guidance (Festinger, 1954; Wheeler, 1991; Wood, 1996; Dijkstra et al., 2008). For example, ‘target’ (SCT) was coded when children referred to the person they compare with, and direction was coded when children mentioned the target’s performance relative to themselves, e.g., ‘he is above me in maths.’ For the second level of analysis codes emerged that were not necessarily part of the theoretical framework. The two levels of analysis provided a balance of findings from both deductive and inductive approaches which are important for theory and practice.

Once codes were assigned, some of the data was quantified by counting frequency of occurrences to examine patterns within the sample and to reveal patterns within and across attainment groups. Children’s narratives and responses were combined with the frequency counts to retain the rich description of the children’s experiences. For example, it was determined that the effect of social comparison was the most frequently discussed theme amongst the children in the study, however the richness of the data provided greater detail about the range of effects, how children of different ability levels recalled these experiences and which experiences were particularly salient. After first-level codes were assigned to all transcripts, a number of interviews were reviewed together with the codes by a researcher independent to the study to check the accuracy of the coding and remove codes that were irrelevant or repeated. Codes that were similar and that were related to one another when then combined to create high-levels codes in tree-node/hierarchical axial format. This allowed for codes to be revised again. The final analysis consisted of a total of 41 codes and six tree-nodes (acknowledgment, frequency, topic, target, direction, and impact).

The study involved analysis of the qualitative interview data and analysis of the quantitative social comparison questionnaire. Scores were taken directly from the questionnaires and entered into SPSS and descriptive statistics were performed. Any written responses to questionnaires were coded and entered. On the rare occasion when an answer was ambiguous (e.g., when the pupil’s handwriting was difficult to read) a second marker (a teacher) independently assessed the response. Given the core component of the study was qualitatively driven to explore and describe the nature of children’s social comparisons, the point of interface of the quantitative component is in the Results narrative as is commonly seen in mixed methods research (Creswell and Plano Clark, 2017).

## Results

Considering the quantitative findings first, 71.1% (*n* = 175) of children acknowledged their engagement in social comparison, with 14.2% (*n* = 35) saying they do not compare and 14.6% (*n* = 36) saying they were not sure. Nearly half of the sample (48.8%, *n* = 120) said they engaged in social comparison infrequently. However, 40.2% (*n* = 99) stated that they would compare themselves to others every day. Friendship was provided as the key reason for selecting a social comparison target (see Table 1).

**TABLE 1:** Reasons for selection of social comparison target.

Reason	N	Percentage %
They are a friend	121	49.2
They are good at the subject	78	31.7
They sit next to me	19	7.7
They are the best in the class	11	4.5
They help me	8	3.3
They are the worst in the class	5	2.0
They are bad at the subject	2	0.8
Other	2	0.8

Maths and Science were the main topics identified by children as being the subjects in which they were more likely to compare (see Table 2).

**TABLE 2:** Social Comparison Subject selection.

Subject	N	Percentage
English	31	12.6%
Maths	94	38.2%
Science	85	34.6%
Sport	24	9.8%
Other*	12	4.9%

*No common theme in ‘other’ category. Categories included art (n = 5), projects (n = 4).

Table 3 displays the information children seek when making comparisons. Using the modified rank order paradigm (see reaction approach above) Table 3 shows the rank of the scores children selected to view. Aggregation of the ranks above the middle (top score, just below top, just above the middle) shows that 84.1% of the pupils compared upwards and choose to gain access to knowledge of scores above their current achievement level. Aggregation of ranks in the top bands (top score, just below top) shows 49.6% of children showed a strong upwards trend, while 44.6% choose to compare close to the middle (just above the middle, just below the middle) indication of horizontal comparison.

**TABLE 3:** Modified rank order paradigm: information children select to compare.

Score choice	% (*n* = 246)
Top score	38.2 (*n* = 94)
Just below top	11.4 (*n* = 28)
Just above middle	34.5 (*n* = 85)
Just below middle	10.1 (*n* = 26)
Bottom score	5.3 (*n* = 13)

Analysis of the scores on the comparative rating question confirmed an upwards direction with the mean score of 3.61 (standard deviation 0.84) indicating that the social comparison target tended to lie between points 3 (“the same as the rest of the class”) and 4 (“better than the rest of the class”). Thus, pupils chose to compare with a target who they thought slightly outperformed the majority of the pupils in the class.

Table 4 shows the effects of comparison where 85.8% of children held positive feelings when they were judged as being better than others, and 96.3% of children held negative or neutral feelings when their performance was worse than others.

**TABLE 4:** Emotional response: judgments being better or worse than others.

Response	Worse than others	Better than others
Positive	3.7% (*n* = 9)	85.8% (*n* = 211)
Negative	58.9% (*n* = 145)	1.2% (n = 3)
Neutral	37.4% (*n* = 92)	13% (*n* = 32)

### Social Comparison Themes

The analysis presented below in Table 5 focuses on the six strands of social comparison that were identified in the data analysis: (1) acknowledgment of social comparison, (2) comparison topic, (3) social comparison target, (4) direction of comparison, (5) self-evaluation, and (6) effects of social comparison. The discussion below considers the qualitative responses of children in different within-class ability groups and highlights of the richness of this data in elucidating the children’s experiences of social comparison. The quantitative description is used to expand certain details of these results.

**TABLE 5:** Overview of themes and sub-themes.

Theme	Sub-theme	Theme	Sub-theme
Theme 1: Acknowledgment	• Disclosure • Awareness • Conscious • Unconscious • Self/Others • Comparative information • Frequency of comparison	Theme 4: Direction	• Upwards • Downwards • Horizontal • Assimilations • Contrasts
Theme 2: Topic	• Curriculum area • Enjoyment • Perceived ability • Intentional • Encountered • Forced • Teachers	Theme 5: Self-evaluation	• Support • Help • Learning • Attainment • Competition • Self-improvement • Misperception
Theme 3: Target	• Friendships • Groups • Seating • Ability • Support • Similarity • Gender	Theme 6: Effect	• Positive • Negative • Double edged • Attitudes to school • Effect on self • Effect on peers • Effect on family

### Theme 1: Acknowledgment of Social Comparison

When first asked, 75% of the children interviewed said they compared their schoolwork with others in their class. A similar response (71%) was evident *via* questionnaire. The following response was typical:

*“yeh*…*I like to see whether people have the same ideas as me*…*I like comparing my work but I don’t always do it” (boy, medium ability-group).*

A difference according to ability groups was observed with 100% of the children assigned to the low-ability groups acknowledging their use of social comparison compared to 58% of those in middle group and 67% in the high group. Not only does this signal a difference in the quantity of social comparisons according to ability grouping but also a difference in the implicit/explicit quality of social comparisons according to ability appeared to be occurring. Although all pupils were able to talk of themselves in relation to others the low ability pupils used comparison more frequently, to a greater extent to judge their own performance and appeared to use social comparison in a more deliberate and explicit way.

Analysis of the interview data of those pupils who failed to immediately acknowledge their use of social comparison appeared to reveal an unconscious/automatic nature of social comparison for children in other ability groups. For example, despite denying that they used social comparison when asked directly, all pupils’ responses to indirect questioning suggested that they did in fact compare. For example, the quotes below shows the different responses to interview questions from the *same* pupil. When asked “Do you compare your schoolwork with others?” the pupil’s response was negative. Yet when replying to the question “How do you judge how good or bad you are at schoolwork”? the same child’s answer showed that they compared often.

*Do you compare your schoolwork with others?*

Carol: *Not normally no*

*How do you judge how good or bad you are at schoolwork?*

Carol: …..*well*….*I know I have done my best and I can’t do any more so I don’t judge my ability on what others have done but I do like to see what others have done especially in science and I compare with them quite often.*

### Theme 2: Social Comparison Topic

What is striking about the pupil’s words quoted above is how the curriculum area in which she used social comparison was identified. Thirty-two of the 36 children interviewed spontaneously identified the specific subject or subjects in which they more likely to compare their work. As in the quantitative questionnaire, Maths and Science were nominated most frequently by pupils. Pupils of different ability groups appeared to be similar in their responses in that not only did the children identify a specific curriculum area but many of the pupils gave a clear reason as to *why* they were more likely to compare in this subject than in others. The reasons given fell into two categories; enjoyment (I like/don’t like the topic) and perceived ability (I am good/not good at it), as illustrated by the quotes below. These findings relate to the Local Dominance Effect (Buckingham and Alicke, 2002; Zell and Alicke, 2010) (see section “Discussion”).

*Sue: I compare sometimes in particular lessons more in English as I am not so good at English and other people are better than me.*

*Paul: Yes, in Art because most people are better than me.*

*James: I like to compare in lessons where maybe I could have done better, my weaker subject.*

*Sally: more in science. It is my strongest subject; I really like it and I want to do well.*

*Tom: I compare in all the subjects I don’t understand. I get confused in English and it’s boring and the teacher is rubbish so I don’t like that subject and I compare in that.*

### Theme 3: Social Comparison Target

Children were asked to nominate one person that they normally compare their schoolwork with (SCT). This comparison is referred to here as an everyday ‘real’ comparison as children are not forced in selecting a preferred comparison given a hypothetical scenario but instead to select a person in their class that they actually do compare with; a real comparison. The nomination of one self-selected SCT allowed further examination of the qualities of self-selected comparisons.

Friendships were a key factor in the choice of SCT in both the quantitative questionnaire (see Table 1) and in the qualitative interviews, regardless of ability group. That said, the interviews revealed that other requirements of the comparison target were sought after in addition to friendship. Pupils wanted their SCT to provide help or be good at a particular subject.

*Julia: she is a friend and she sometimes gets it right better than others.*

*Harriet: she is a close friend.....she struggles at maths and English but she is better at science.....well I think so. We do work well together.*

*Sam: he is a friend and he listens to me and says what I got wrong, others like Ben just mess around.*

Whilst pupils were easily able to identify the SCT they compare with the most, in the interview’s children’s ‘free responses’ provided evidence of comparison with more than one SCT and occasionally different SCT’s for different topics. No child referred to more than three SCT’s. Seating/table assignment was a key influence on self-selection of the SCT as the quotes below illustrate:

*Sarah: I compare to the person I sit next to who is a friend. I compare more if I sit next to a friend. Only if I get stuck will I compare with someone who is not a friend.*

*Jack: in science we can sit next to whoever we like*…*If I sit next to a friend I am more likely to compare although if I don’t understand then I’d compare with someone in the group even if they are not a friend.*

While ‘free choice’ self-selection of SCT was preferred by all children, it appeared that to some extent the SCT is a forced option rather than a deliberate choice in the groups. Many pupils spoke of being allocated places by their teachers and as a result having to compare with the person sitting next to them. The forced comparisons were associated with lower frequency of comparisons as illustrated in the quotes above, with pupils saying they only compare if they are struggling and more likely to compare if they can choose a friend to compare with.

### Theme 4: Direction of Comparison

As previously discussed, the direction of comparison can be upwards, downwards, or horizontal. That is, pupils can compare themselves with someone above their ability level, below their ability level or with someone at the same level of ability. Different methods were used to gauge direction of comparison. The reaction approach used comparative rating to ask pupils to rate themselves against others and thus explored pupil’s perception of relative ability. It is important to bear in mind that this is not actual ability, but perceived ability as judged by each pupil. This method suggested that pupils compared themselves upwards (69%) or horizontally (25%) (25 pupils said they were the same as the rest of the class, 9 said better and 2 said worse). The quantitative questionnaire confirmed this upwards/horizontal finding. The mean score of 3.61 indicated that on the whole pupils said their comparison target was between “the same as the rest of the class” (point 3) and “better than the rest of the class” (point 4).

The selection approach confirmed the upward direction of comparison using the adapted rank order paradigm pupils were given a hypothetical scenario and asked to select a SCT based on rank (see quantitative measures for detailed description). When presented with this scenario 31 of the 36 children (86%) chose to view a score above their own (above the middle) and 5 chose to view a score below their own (3 of which were from the low ability group). Similar results were found quantitatively with 84% choosing to view above the middle rank (see Table 3). Due to the scenario forcing children to choose a different rank to the one they occupied there was no specific measurement of horizontal comparisons in this approach, however, aggregating scores around the middle revealed that 44.6% choose to compare close to the middle (indicating horizontal comparisons) 49.6% of children choose top bands indicating a strong upwards preference. Thus closer inspection suggests preference for upwards or horizontal social comparison.

The narration approach confirmed the upwards/horizontal direction with approx. half of the children comparing upwards and half horizontal. When asked about the ability level of their SCT, 47% (*n* = 17) of those interviewed said that their SCT was better than most of the class and 53% (*n* = 19) said that the SCT was the same as the rest of the class. Ability group did make a difference here. The SCT for low and high ability groups tended to be the same as the rest of the class whereas the medium ability groups tended to compare themselves with someone that they perceived to be better than the rest of the class. In other words, the direction of comparison was horizontal for the high and low abilities, and upwards for the medium ability children.

### Theme 5: Self-Evaluation Performance Judgments

As discussed above, all children judged their SCT to be better than or the same as the rest of the class. These performance judgments not only provided an indication of the direction of comparison but also allowed one to explore how pupils judged performance. Three reasons were given by the pupils as to *how* they were able to gauge the ability of the SCT namely: knowledge of scores, observation of pieces of work, and viewing class discussions participation such as public questioning.

In terms of their own ability in relation to the rest of the class, the high ability group tended to judge themselves in the top two quarters of the class, the medium group judged themselves to be in the middle two quarters and the low ability group judged themselves to be across the 2nd, 3rd, and 4th quarters. When asked *how* pupils gauge their *own* performance the response was varied and less certain than when asked about the SCT. Half the pupils couldn’t seem to answer the question and gave vague responses. This is perhaps not unexpected. Pupils gauged others’ academic ability on what they saw and heard in the classroom and as such they understood that this was how they judged others’ ability. In contrast pupils could not state how they came to know things about themselves. By the age of 11 children would have had a wealth of experiences on which to base their self-perceptions and perhaps found it difficult to select one or two reasons. This occurred throughout the interview. Pupils were happy to provide a judgment regarding themselves but were either slow or unable to express how they came to that judgment.

*Paul: I don’t struggle but I am not really, really good.*

*Sue: I don’t know. I know I am ok and so are the other people in my class.*

To some extent the above implies that children are overtly engaging in social comparison and thus can describe the reasons for such a judgment but are unaware of the impact it plays upon themselves. The inference of deliberate comparison with others was confirmed when the discussion moved away from general judgments of ability to more specific judgments of pieces of work. Pupils were asked to think how they might judge their work in class if they were given a task to complete today. All pupils offered a more detailed account with pupils either describing how the teacher would say or do things that would inform them of their performance, how they would use the content of their own work to judge their performance or how they would compare their work with others to judge the quality of their work. Children in the low ability groups were more likely to refer to comparison with peers (83%) than children in the medium (42%) or high ability (33%) groups.

### Theme 6: Effects of Comparison

All pupils identified the emotional impact of social comparison. Pupils’ responses fell into three categories: dual emotions, confidence or negative emotions. Dual emotions refer to the differing emotions, both positive and negative, because of social comparison. Approximately 40% of those interviewed discussed the contented, pleased reaction gained from superior performance compared to a comparison target (SCT) and the disapproving, anxious feelings exhibited as a result of inferior performance. Moreover, as the quote below highlights children are using the SCT as a way to judge the standard of their schoolwork as social comparison theory (Festinger, 1954) posits:

*William: Ok*…*if mine is just as good as his then I feel pleased because I know I have reached a good standard. If his is better than mine I feel* jealous …I think that I need to try better next time.

*Scott: When I get a better grade, I feel sort of proud of myself, not like boastful or anything but happy.*

The quantitative results highlight that comparing favorably evokes positive emotional responses in most children (see Table 4). 84% of children surveyed said they would feel positive if their work was better than their peers. For 14 of those interviewed, social comparison with their classmates produced only feelings of increased confidence. These pupils tended to mention the confidence and peace of mind gained through social comparisons in that they reduced the uncertainty involved in completing a piece of work at school.

*Mark: More confident I suppose that he has the same ideas.*

A small number of pupils (17%: 6 of the 36 interviewed) only saw the negative aspects of social comparison and discussed negative emotions exclusively. In contrast in the quantitative questionnaire only 1% of children reported negative responses to both upward and downward comparison (Table 4). The qualitative interviews did aim to unpack how the children experienced social comparisons and thus this difference can be explained methodologically. All of these pupils who spoke only of negative emotions from social comparisons were from the low ability group. Those children assigned to low ability groups experienced more feelings of negativity as a result of their social comparisons than those in medium or high groups.

*Amy: It makes me feel stupid, jealous and frustrated.*

*Max: I just hate myself for being so stupid.*

As the quote above illustrates, for some children within the low ability groups social comparison with others can be a terrible experience.

## Discussion and Implications

The findings presented here provide an indication of the vital role social comparison plays in the primary school classroom. Social comparison was found to be a highly prevalent practice experienced by *all* pupils regardless of ability group. These findings support previous research which shows that the classroom environment is the perfect place for comparisons (Dijkstra et al., 2008) given the extensive source of social comparative information (Levine, 1983; Buunk et al., 2005) and the evaluative atmosphere (e.g., Pepitone, 1972; Levine, 1983). While all children did compare, a difference according to ability group was evident with children assigned to the low-ability groups using social comparison more frequently, consciously and deliberately. The reason for such a finding could lie in the underlying motivation for comparison. Researchers such as Kruglanksi (1989) and Wheeler et al. (1997) have posited that uncertainty motivates comparison. Indeed, most of the pupils in the current study identified level of perceived ability as being one of the key motivators toward comparison. Individuals seek social comparison in situations when other routes to self-assessment are not feasible to reduce uncertainty and gain valid appraisals of themselves. It is possible, therefore, that children working in low ability groups actively seek comparative information in order to reduce uncertainty whereas pupils in high ability groups are either more certain of themselves or are able to use other forms of self-assessment. Indeed this finding resonates with research which has found that subordinate and dominate group status influenced the impact of social comparison on self-esteem and that dismissal of comparison information as a mechanism of self-protection was reserved for those of dominant groups (Martinot and Redersdorff, 2003, 2006). Thus, high ability group pupils as members of the dominant group, unlike the low ability subordinate group, appeared able to dismiss social comparative information.

Exploring children’s acknowledgment/awareness of social comparison, the present study found that a large majority of children acknowledged their use of social comparison, with similar results exhibited on both quantitative and qualitative measures. Whilst it was not surprising that all children compared, the acknowledgment of such engagement was unexpected given previous research which had indicated that pupils were reluctant to admit to comparing themselves with others (Ruble, 1983). However, this may be explained methodologically. Previous research on social comparison has tended to involve quantitative measures only and/or laboratory settings (Wood, 2000). It is, therefore, possible that the informal, non-judgmental nature of the individual qualitative interviews conducted within the school setting in the present study may have contributed to this finding. Indeed, particular thought was given to the framing of the question regarding social comparison since pilot testing the meaning of comparison had revealed that pupils of 10 and 11 years of age did not differentiate between “comparison” and “copying.” Copying is frowned upon in school and thus admitting to such an act would tend to have negative consequences. To avoid this, questionnaires and interviews included an everyday example of social comparison and a reminder that it did not refer to copying. This meant that pupils were able to discuss the act of comparing their work with others with a clear understanding of the meaning of comparison. This may have been an important first step for the children, enabling them to understand and discuss when, where and with whom they compared their work and the effects of such comparisons.

It should be noted that pupils who didn’t immediately acknowledge their engagement in social comparison did so after a ‘warm up’ to the interview. For example, the same child would initially report not comparing with peers and then later in the interview describe their frequent use of social comparison. Reconciling this incongruence, it may be a social desirability response as discussed earlier or it could potentially signal an unconscious element of social comparisons. As Wolff et al. (2020) note unconscious social comparisons have been reported in previous research (e.g., Mussweiler et al., 2004; Alicke and Zell, 2008) yet research is extremely limited to such an extent it is not mentioned in Gerber et al. (2018) recent review, thus further research on unconscious social comparisons is warranted. Alternatively, the ability for the children to ‘warm up’ to the interview could be the reason for the incongruence in response. As discussed above, a key strength of the qualitative aspect of this study was that it enabled exploration of social comparison within different ability groups in a safe environment for children to express themselves, without danger of influence of other children. This ‘safe space’ could have allowed children to overcome any initial reluctance to and feel comfortable to discuss their engagement in social comparison. This would be very reasonable given the negative emotional impact social comparisons can have on a child. Research conducted in naturalistic settings emphasizes understanding the context of the study and tailoring the design (Argio et al., 2019). The qualitative aspect of this study appears to have fulfilled this by allowing discussion and freedom for children to fully understand and engage in the topic, enabling us to gain greater knowledge about the nature of real comparisons in the classroom.

Findings in relation to *who* children compared with (the SCT) concur with previous research that has emphasized the salience of SCT’s (Blanton et al., 1999; Huguet et al., 2001; Liem et al., 2013). The findings here show that the SCT was chosen deliberately by pupils according to their qualities as a friend and characteristics which would render them helpful, supportive, or academically useful. Friendships thus are very important yet often overlooked or discounted when teachers form ability groups. Indeed, in the classroom environment children couldn’t always choose who they compared with and grouping, table assignment and seating impacted comparison frequency and comparison outcome. If children couldn’t compare with their self-selected/chosen SCT then the frequency and motive for comparison altered and consequently the impact of the comparison changed. This is important as it shows the influence teachers can have on social comparisons through grouping practices. Children’s ‘free’ responses (in response to open questions about social comparison) tended to focus on local comparisons with one or two friends. They didn’t actively seek comparisons with large number of peers or groups of children, and at no time did any pupil mention comparisons with the class. Children placed great emphasize on actively seeking social comparison with a select small number of other children and these social comparisons were very important to the children. These findings support the Local Dominance Effect (Buckingham and Alicke, 2002; Zell and Alicke, 2010) which shows that comparisons with few individuals has a greater influence on self-assessments than comparisons with larger aggregates. Indeed, the current study found that whilst pupils did talk about *what* they compare and *why* they compare, *who* they compare with was discussed by pupils far more and thus appeared to exert a greater influence on the child.

Examining *what* pupils are comparing, the present study found that children readily identified the curriculum subject they were more likely to compare and give reasons for their motivation to compare. These findings indicate greater reliance on dimensional comparison than social comparison. According to dimensional comparison research (Möller and Marsh, 2013; Strickhouser and Zell, 2015; Wolff et al., 2018) individuals compare their achievements or abilities in one subject (e.g., science) to their achievements or abilities in other subject (e.g., maths). Dimensional comparisons are made between domains/subjects and social comparisons within the same domain. The findings here concur with that in found other studies of dimensional comparison with children feeling better about (liking) a subject in which their performance is superior. Moreover, a recent research on academic enjoyment (Boliver and Capsada-Munsech, 2021), determined that liking school subjects in ages 7–11 years was vital for academic achievement.

Overall, the findings here showed a preference to compare upwards, resonating with previous research in classrooms (Blanton et al., 1999; Huguet et al., 2001; Gerber et al., 2018). However, there was a strong (and near equal) preference for horizontal social comparison. The direction of comparison is important for children’s self-evaluation. As discussed in the introduction, according to social comparison theory (Festinger, 1954) people use other people as the standard to judge themselves against. Achieving a grade C could be viewed as being very good if one’s peers achieved grade E, but poor if one’s classmates achieved grade A. If a child compares ‘upwards’ with someone above themselves (e.g., the grade A classmate), they can either think they are not as good as the other child or identify with the other child and think they can improve. The findings here showed a convergent of results across different measures, when looking at the aggregates, confirming a tendency to compare upwards. However, the preference for horizontal comparisons was also strong and the interviews showed a slightly higher preference for horizontal over upwards comparison (horizontal 53%/upwards 47%). Unpacking this further we see a difference by ability group, with the high and low abilities engaging in horizontal comparisons, and the medium ability upwards comparisons. Of course, it is possible that this finding is due to reporting bias. High ability pupils may not have felt comfortable labeling their comparison target “worse” and low ability pupils may not have wanted to acknowledge their inferiority by admitting that their SCT was “better.” That said, given the ‘safe space’ of the interviews discussed above this seems unlikely.

Drawing on previous research on groups (Martinot and Redersdorff, 2006; Alicke et al., 2010; Martinot et al., 2020), Social Identity Theory (Tajfel and Turner, 1986) and self-evaluation maintenance theory (SEM) a more likely explanation explored here is that group membership is influencing children’s self-perceptions and psychological protection is at play impacting social comparisons. According to Social Identity Theory (Tajfel and Turner, 1986) individuals can think of themselves in terms of an ‘individual’ self or their ‘group’ self. This can vary according to context and thus as individual can exhibit different aspects of the self in different settings. In some circumstances people may prefer to compare themselves with members of the same group (intragroup) especially if they compare favorably to the group as a whole to such an extent that they ignore any out-group standards and base their self-evaluation on their position within the group (Major, 1994). Indeed, Alicke et al. (2010) found that simply dividing students into arbitrary groups produced a tendency for students to focus on local information to categorize their standing in a group which was used for self-evaluations. In the present study, the high ability pupils as members of the dominant group, are motivated to retain their position, they can dismiss comparison information and as there isn’t a higher group, they compare horizontally with other group members. The medium ability aspires to be in the dominant group, assimilating to be like those above them and thus compare upwards. The low ability children, as members of the subordinate group cannot compare downwards as they occupy the lowest rank. The question is why aren’t these children comparing upwards? Self-evaluation maintenance theory (Tesser, 1988) assumes that all individuals try to maintain a positive self-evaluation and utilize alternative ways in which social comparative information is received and processed. While upwards comparisons can promote growth and learning and thus have positive impact, if an individual focuses on the contrasts (differences) between them and the target it can lead to feelings of inferiority, jealousy and inadequacy (see earlier discussion). Given the impact on emotional impact of social comparison found in this study, it is posited that the low ability pupils are actively seeking comparative information horizontally in order to protect themselves against the negative impact of ‘unattainable’ upwards comparisons. This resonates with research on psychological disengagement mechanisms with adolescents where students from disadvantaged groups discount or and devalue grades or unhappiness due to a unfavorable social comparisons to protect themselves from negative outcomes (Martinot et al., 2020).

There is no disputing the emotional/psychological impact of social comparisons. All pupils were to some extent affected by comparisons with their peers, although this was more extreme and more frequent for those assigned to low ability. The mixture of emotions experienced by pupils through social comparison showed the turmoil and insecurity that some pupils faced on a daily basis. Having a child within the interviews admit to hating themselves because of comparisons with their classmates is an illustration of the profound negative impact it can have on a child’s life yet it appeared that this was accepted as part and parcel of the social interaction that occurred within ability groups.

### Limitations

Despite the strength of the mixed methods in the present study, there were limitations. Only 12 primary schools in similar geographical location were included in this study which narrows the cultural diversity and generalizability of the study. The qualitative, ecologically valid, naturalistic aspect of this study is its strength, designed to explore the nature of social comparisons in the classroom. The quantitative data enhanced the descriptions, enabled comparisons, and illustrated and confirmed the qualitative findings yet could have added more depth had the quantitative component been given more equal weight in the design. Moreover, the anonymous/de-identified design of the quantitative questionnaires (ethical requirements) did not permit direct matching to individual qualitative data which would have provided additional triangulation to support findings. Adapting the interview design so that the questionnaire is completed during one of the interviews would overcome such a constraint. Alternatively, a sequential mixed methods design may have merit. Having the quantitative first and then using interviews to explain the data in a sequential explanatory design would allow selection of children on the basis of their responses.

Further limitations of the quantitative measures involve the use of rank order scenario. While it captured, upwards/downwards direction of social comparison and near/extreme ranks, the forced method required children to choose a different rank to the one they occupied which didn’t permit measurement of horizontal/lateral social comparison. Given that horizontal comparisons were salient in the qualitative data, being able to capture this quantitatively would be beneficial. As discussed earlier, the measurement of social comparison is complex and one of the key criticisms of forced comparisons is that they don’t relate to the ‘real world’ (Wood, 2000), with researchers cautioning against their generalization (Dijkstra et al., 2008; Gerber et al., 2018; Boissicat et al., 2020). Thus, the limitation of quantitative forced comparisons highlighted here concurs with previous research and leads us to advocate for mixed methods and qualitative research to build on the existing quantitative literature and forward the field.

## Conclusion

The findings presented here provide evidence of the vital role social comparison plays in the primary school classroom. Social comparison amongst 10–11-year-old children was found to be a highly prevalent daily aspect of classroom life experienced by *all* pupils regardless of ability group. Children engaged in social comparisons at different levels, yet the importance of friendships and local comparisons (where children actively sought comparisons with a very small number of friends) were far more important to children (the Local Dominance Effect). The fluency with which children discussed comparisons with peers was staggering and tends to suggest a strong prevalence of this of method for self-evaluation. This was particularly salient for children assigned to low-ability groups who were more vulnerable to the negative effects of social comparison.

While all children compared, children assigned to low-ability groups used social comparison more frequently, consciously and deliberately, actively seeking horizontal intra-group comparisons. In doing so they avoided upwards comparisons thereby protecting themselves from unfavorable comparisons with others. One explanation explored within this paper is that group membership/group standing influences children’s self-perceptions and self-evaluations to such an extent that psychological protection mechanisms kick in and alter/impact social comparisons processes. Yet it is forwarded here that while these strategies may provide psychological ‘protection’, they may also be the roadblock in the path to better outcomes for such children. Ability groupings mean that pupils are forced to compare with same level pupils. For low ability children, whilst this may ‘protect’ children from negative comparisons and self-evaluations, it means they miss out on comparisons with more able pupils. If we accept that social comparisons can facilitate learning, encourage growth and self-improvement, then these children are missing out on the very practices that could support them. Seeing other children solve problems, explain their thinking and learning through shared discussion is vital and so limiting these opportunities for children appears to be misguided.

With some investment in understanding how better to group children (or not group children) and how to remove psychological roadblocks, teachers can set up classroom practices where children don’t have to protect themselves. You only have to look at the affective reactions to social comparisons in this study to see the vital role they play in children’s well-being and emotional stability. All pupils were to some extent affected by comparisons with their peers, negative and positive, although the negative impact was more extreme and frequent for those assigned to low ability groups. Pupil–pupil interaction in the classroom that leads a child to say they hate themselves clearly needs to be addressed.

Teacher education and professional development on this topic could help teachers to identify and support children that rely on self-evaluations of performance based on social comparisons and redirect them to teacher appraisal and feedback. Moreover, given that self-perceptions are related to actual performance, teachers should be alerted to the implications of social comparison for motivation, engagement and achievement. The argument presented here is that teachers need to put a greater emphasis on pupil–pupil interactions and social comparisons when forming within-class ability-groups. The collaborative nature of classrooms today means greater emphasis on talk and discussion for learning and thus greater availability of social comparative information. The groups children are assigned to influences what information children hear or see about their classmates and what teacher input/time they are allocated. Grouping practices including peer–peer interaction, seating, table allocation needs to be considered in terms of social comparison opportunities. Forced comparisons (comparisons chosen by the teacher as opposed free choice self-selected comparisons by the child) altered the frequency and motive for comparisons and thus self-evaluations. Appropriate planning by teachers of these interactions is advocated which take account of the extent to which pupils of different ability groups process and utilize social comparative information and consider grouping children in different ways – so that children work in a range of groups and in particular consider friendships as a way to group children in primary classes. Thus, there is significant value in understanding more about how teachers can harness the power of social comparison to enhance outcomes for children. To achieve this further research is needed to understand the everyday social comparisons children make in the classroom. This study has provided evidence for the importance of capturing qualitative data and creating ‘safe spaces’ and including ‘warmups’ in the interview design. Thus, capturing social comparisons utilizing qualitative and mixed research methods in natural environments is advocated given the depth of understanding it affords.

## Data Availability Statement

The raw data supporting the conclusions of this article will be made available by the authors, without undue reservation.

## Ethics Statement

The studies involving human participants were reviewed and approved by the University of Cambridge. Written informed consent to participate in this study was provided by the participants’ legal guardian/next of kin.

## Author Contributions

The author confirms being the sole contributor of this work and has approved it for publication.

## Conflict of Interest

The author declares that the research was conducted in the absence of any commercial or financial relationships that could be construed as a potential conflict of interest.

## Publisher’s Note

All claims expressed in this article are solely those of the authors and do not necessarily represent those of their affiliated organizations, or those of the publisher, the editors and the reviewers. Any product that may be evaluated in this article, or claim that may be made by its manufacturer, is not guaranteed or endorsed by the publisher.
